# Correlation Between Infectivity and qRT-PCR Values for Murine Norovirus Recovered from Frozen Berries

**DOI:** 10.1007/s12560-025-09668-w

**Published:** 2025-10-31

**Authors:** Daniel Plante, Julio Alexander Bran Barrera, Maude Lord, Jennifer Harlow, Irène Iugovaz, Neda Nasheri

**Affiliations:** 1 Microbiology Laboratory, Regulatory Operations and Enforcement Branch, Health Canada, 1001 St-Laurent Street West, Longueuil, QC J4K 1C7 Canada; 2 National Food Virology Reference Centre, Bureau of Microbial Hazards, Food Directorate, Health Canada, 251 Sir Frederick Banting Driveway, Ottawa, ON K1A 0K9 Canada; 3 Department of Biochemistry, Microbiology and Immunology, Faculty of Medicine, University of Ottawa, Ottawa, ON Canada

**Keywords:** Norovirus, Infectivity assay, qRT-PCR, Frozen berries

## Abstract

Human norovirus (HuNoV) is the leading cause of acute gastroenteritis globally, with frozen berries frequently implicated in foodborne outbreaks. Current surveillance relies on quantitative reverse transcription PCR (qRT-PCR), which cannot differentiate between infectious and non-infectious viral particles, complicating risk assessment. This study is aimed to establish the minimum viral load on frozen berries detectable by qRT-PCR that corresponds to infectious virus, using murine norovirus (MNV) as a surrogate for HuNoV. Frozen raspberries were artificially inoculated with serial dilutions of MNV (7.1–1.0 log PFU/25 g) and processed using the ISO 15216:2017 method. Infectious virus was quantified by plaque assay, and viral RNA was detected by qRT-PCR. The limit of detection (LOD) for cell culture was 3.1 log PFU/25 g, whereas qRT-PCR extended sensitivity to 1.0 log PFU/25 g (Ct value at 36.7 ± 0.6), representing a 2-log difference. Recovery rates for infectious virus exceeded the ISO 15,216 minimum threshold (1%), and PCR inhibition was negligible. We next examined the extraction efficiency for both infectious MNV and its genetic material from frozen strawberries at inoculation levels higher than the LOD, and observed that the viral recovery from frozen strawberries is very similar to viral recovery from frozen raspberries with no significant differences between them. The disparity between LODs indicates that a substantial proportion of MNV genomes detected by qRT-PCR do not represent infectious particles, aligning with previous findings that one PFU may correspond to multiple genome copies. Given that many surveillance studies report high Ct values (> 35), our data suggest that such detections may not indicate viable virus, underscoring the importance of contextualizing qRT-PCR results with epidemiological evidence. These findings highlight the need for cautious interpretation of surveillance data, particularly for public health decision-making.

## Introduction

Human norovirus (HuNoV) is the leading agent of acute gastroenteritis, causing millions of infections annually (Havelaar et al., 2015). Although characterized as mild infections, in risk groups, such as children under five, elderly or immunocompromised people, norovirus infections can lead to severe outcomes (Bartsch et al., 2020). In the past two decades, numerous HuNoV outbreaks have been attributed to contaminated berries in particular frozen berries (Bozkurt et al., 2021; Nasheri et al., 2019). The most frequently implicated berry types are strawberries and raspberries as there is evidence that norovirus is very adherent to the surface of these berries (Tian et al., 2011; Trudel-Ferland et al., 2021).

In order to reduce the risk of foodborne HuNoV infections, food safety authorities implement regulations and surveillance programs to monitor compliance to the regulations that utilize standardized molecular tests, such as quantitative reverse transcription polymerase chain reaction (qRT-PCR), to assess the levels of HuNoV contamination in high-risk foods (ISO, 2017; Lowther et al., 2019). However, detecting HuNoV in food samples is complicated, mainly due to the absence of routine in vitro cultivation methods to propagate the virus from naturally-contaminated samples. The general approach for detection of enteric viruses in food samples consists of two major steps: (i) sample preparation (virus concentration and nucleic acid extraction); and (ii) detection and quantification of viral genome (usually by qRT-PCR). In this approach, viral detection is based on the amplification of a fragment of the viral genome, and is unable to distinguish between infectious and non-infectious viral particles (Jaykus et al., 2025).

This approach, combined with epidemiological evidence has been implemented for outbreak control and post outbreak analysis (Papafragkou et al., 2025; Raymond et al., 2022; Summa et al., 2024). However, qRT-PCR results in the absence of epidemiological data, pose a significant challenge for comprehensive risk assessments, as multiple studies have shown that food samples that are positive by qRT-PCR might not contain infectious virus. For example, in a human challenge study, none of the 20 participants that consumed berries contaminated with 120–252 genome copies per gram reported any symptoms (Eshaghi Gorji et al., 2021), suggesting the common presence of non-infectious virus or viral RNA fragments. Data regarding the correlation between viral genome copy detected in food samples and human illness is scarce, but it appears that food samples associated with outbreaks and illnesses, have higher viral load compared to the surveillance studies that did not result in human illnesses, for example, one study on naturally contaminated oysters demonstrated that the samples that were associated with human illnesses had approximately one log higher viral load compared to the ones that were not (Lowther et al., 2012). Therefore it is important to know the minimum level of viral genome copy number in food samples that could lead to infection. The aim of this study is to determine the minimum viral titer from frozen berries detected by qRT-PCR that would lead to successful norovirus infection. The current human intestinal enteroid system (HIE) for HuNoV cultivation requires high inoculum levels and does not produce consistent results for viral loads that are often found in naturally contaminated berries (2–3 log genome copies per g (Wales et al., 2023). For this reason, we used a wide range of concentrations of murine norovirus (MNV), as a surrogate for HuNoV, to inoculate frozen raspberries and strawberries in order to determine the lowest inoculum levels that would lead to a successful MNV replication and quantification in cell culture.

## Materials and Methods

### Cells and Viruses

Murine BV-2 cells were maintained in Dulbecco’s Modified Eagle Medium (DMEM; GIBCO, Cat. No. 12800-058) supplemented with 2 mM L-glutamine (GIBCO, Cat. No. 25030-081), 100 U/mL penicillin,100 µg/mL streptomycin (GIBCO, Cat. No. 15140-122), 3 g/L sodium bicarbonate and 10% (v/v) fetal bovine serum (FBS; GIBCO, Cat. No. 16140-071).

MNV-1 was kindly provided by Dr. Virgin, Washington University School of Medicine, St. Louis, MO. MNV-1 stock were prepared as previously described (Nasheri et al., 2021). Briefly, MNV-1 was propagated in murine BV-2 cells. When cytopathic effects reached approximately 80% as determined microscopically, the cells were lysed with two cycles of freezing at −80 °C and thawing at room temperature. The virus suspension was clarified by centrifugation at 400 x *g*, 20 min then passed through a 0.22 µM filter to remove cell debris and stored at −80 °C until use. Virus titer was determined by plaque assay conducted as described previously (Fallahi & Mattison, 2011).

### Inoculation of Frozen Berries

Frozen berries (raspberries and strawberries) were purchased from local grocery stores (Longueuil, QC). Portions of 25 g were weighed in petri dishes and allowed to thaw at room temperature. The berries were inoculated with 100 µL of MNV suspension dispersed in multiple droplets. The inoculum concentrations ranged from 7.1 to 1.0 log PFU, with additional concentrations between 4.1 and 3.1 log PFU as the limit of detection was expected to be in this range (Wales et al., 2023). The samples were placed in a biological safety cabinet until the inoculum was visibly absorbed (approximately 30 min) then transferred into mesh filter bags and processed as described below.

### Virus Recovery with ISO 15216 Methodology

Viruses were recovered as described in ISO 15,216 :2017 (ISO, 2017). Briefly, 40 mL of Tris-Glycine-Beef extract buffer (TBGE) with 30 units of pectinase from *A. niger* was poured into the bags and incubated at room temperature for 20 min under constant agitation at 60 rpm. pH was adjusted to 9.5 with NaOH at the beginning of the incubation and every 10 min thereafter. The liquid was recovered in 50 mL screw-cap tubes, clarified by centrifugation at 10 000x*g*, 30 min, 4 °C and the pH was lowered to 7.0 with HCl. The volume of rinsate was measured and 0.25 volume of 5X PEG/NaCl [500 g/L PEG 8000, 87 g/L NaCl] was added, followed by incubation at room temperature for 60 min at 60 rpm constant agitation. The tubes were then centrifuged as mentioned above and the supernatant was gently discarded. As the pellets were very loose, residual supernatant was recovered and discarded with a second round of centrifugation. The pellets were resuspended in 1 mL Phosphate Buffered Saline (PBS) and used for plaque assay. For RNA extraction, an equal volume of chloroform: butanol was added to the virus suspension. The aqueous phase was recovered by centrifugation at 10 000x*g*, 30 min at 4 °C and extracted with the NucleoSpin RNA virus funnel kit from Macherey-Nagel as per manufacturer’s instructions. Final elution volume was 100 µL.

### qRT-PCR Detection

The extracted RNA was tested by qRT-PCR as published previously (Bae & Schwab, 2008; Plante et al., 2021), using undiluted and 1/10 diluted RNA.

### Plaque Assay

The viral suspension was serially diluted in Dulbecco’s Modified Eagle Medium (DMEM) without amino acids and without Fetal Bovine Serum (FBS) and used for plaque assays. BV-2 murine microglial cells were seeded in 12-well plates at a density of 8 × 10⁵ cells per well in 2 mL of DMEM and incubated for 24 h at 37 °C with 5% CO₂. On the day of infection, three wells per dilution were inoculated with 150 µL of viral suspension and incubated for 1 h at 37 °C, with gentle rocking every 10 min to facilitate viral adsorption. The inoculum was then removed, and the cells were washed 2 times with PBS (8 g/L NaCl, 0.2 g/L KCl, 1.44 g/L Na₂HPO₄, 0.24 g/L KH₂PO₄, pH 7.4). An overlay medium consisting of equal parts 1.4% agarose and 2× DMEM was applied (2 mL per well), and plates were left at room temperature for 15–20 min to allow the overlay to solidify. Plates were then incubated for 48 h at 37 °C with 5% CO₂.

After incubation, the cells were fixed with 2 mL of 3.7% paraformaldehyde for a minimum of 4 h at room temperature. The agar overlay was then removed and the wells were stained with 0.1% crystal violet for 20 min. Plates were rinsed with tap water and air-dried. Plaques were counted manually, and viral titers were calculated as plaque-forming units per milliliter of inoculum (PFU/mL), then converted to PFU per gram of the starting sample.

### Recovery Rate Calculations

The recovery rate for each method was determined as the ratio between the recovered viral titre (PFU) from each sample to the inoculated viral titre (PFU) $(recovered\:PFU/inoculated\:PFU)\times\:100$.

### Determination of the Limit of Detection

Each sample was artificially inoculated with decreasing viral titers, in triplicate. At T_0_, which is the time of sample processing immediately after inoculation, the virus was extracted and assayed by plaque assay as described above. The plaques were counted for each inoculation titer and the results were analyzed to determine the lowest titre for which plaques were still obtained.

### Statistical Analysis

Statistical analysis was performed using GraphPad Prism v9.0 (GraphPad Software) and the one-way ANOVA method for extraction efficiency comparisons at each inoculation level. Analysis of comparisons of viral extraction from frozen berries at two inoculation levels was performed by t-test.

## Results

### Determining the Limit of Detection (LOD) on Raspberries

**Fig. 1:**
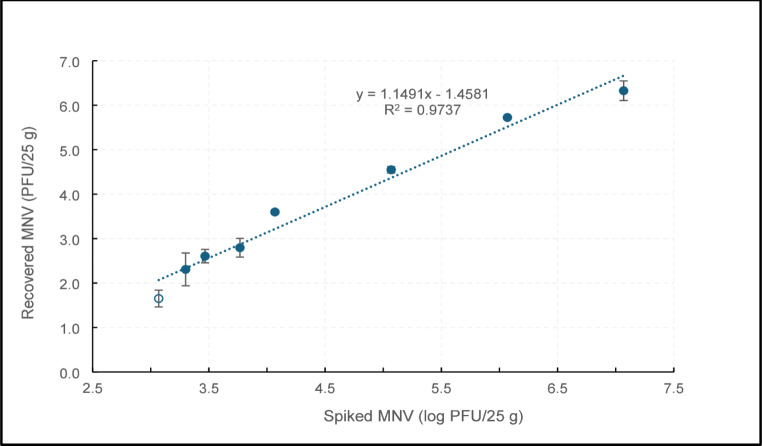
Determining the limit of detection (LOD) for infectious MNV extracted from frozen raspberries. Each data point is the average of 3 replicates with error bars showing standard deviation. The empty circle demonstrates partial recovery

**Fig. 2:**
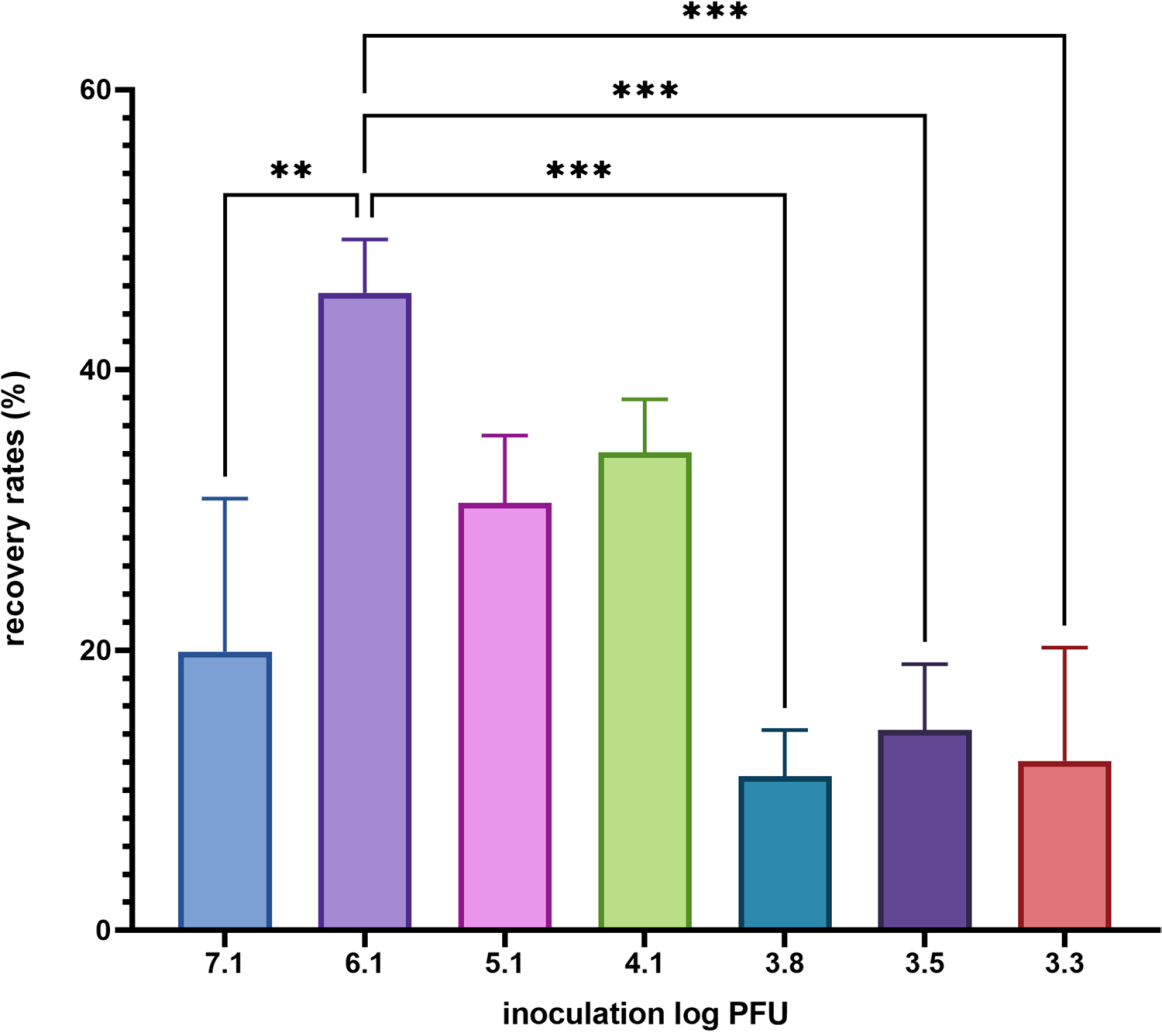
Recovery rates by cell culture for infectious MNV extracted from frozen raspberries. The data is the average of 3 replicates and error bars represent standard deviation. **p ≤ 0.01, and ***p ≤ 0.001 calculated by one-way ANOVA

In order to determine the LOD for both plaque assay and qRT-PCR, frozen raspberries were inoculated with varying concentrations of MNV from 7.1 to 1.0 log PFU/25 g and subjected to viral extraction procedure, using the ISO 15,216 protocol. As shown in Fig. 1, the number of plaques and calculated virus recovery followed a linear relationship (R2 = 0.974) with the inoculated MNV concentration down to inoculation level of 3.1 log PFU, where 2 out of 3 replicates yielded plaques. At inoculation level of 2.1 log PFU, no plaque was detected, therefore the LOD for the detection of infectious MNV on raspberries, defined as the inoculated concentration that yields fractional recovery, was determined to be 3.1 log PFU/25 g. The recovery rates for infectious MNV across the inoculation levels above the LOD varied between 10% and 45% as demonstrated in Fig. 2. Overall the recovery rates for extraction of infectious virus across the inoculation levels were significantly higher than the acceptable threshold, which was 1%. The highest recovery rate was achieved at 6.1 log PFU inoculation level and the difference is statistically significant compared to other inoculation levels.

Viral nucleic acid was detected by qRT-PCR from samples inoculated in parallel to those used for cell culture. In all qRT-PCR experiments, undiluted and 1/10 diluted RNA were tested, and undiluted RNA produced better results (Table 1). Since the Ct value difference between the undiluted and 1:10 diluted samples across all inoculation levels is much greater than 2, the inhibition rate is considered negligible (Table 1) (ISO, 2017). Dilution of RNA might alleviate some potential PCR inhibition if present but, as Ct values were never improved by dilution, this practice was ultimately detrimental to the estimation of LOD in a qualitative detection context. Therefore, the results from undiluted RNA were retained for analysis.

**Table 1:** Viral titers obtained from inoculation of frozen raspberries by both plaque assay and qRT-PCR. 1:1 and 1:10 indicate the ratio for RNA Dilution prior to qRT-PCR

Inoculation	Cell culture	qRT-PCR (Ct values)	
log PFU	log PFU	1:1	1:10
7.1	6.3 ± 0.2	17.4 ± 0.3	19.9 ± 2.3
6.1	5.7 ± 0.04	17.5 ± 0.6	23.8 ± 1.0
5.1	4.5 ± 0.07	20.6 ± 0.5	27.4 ± 1.1
4.1	3.6 ± 0.05	25.2 ± 0.2	30.1 ± 1.2
3.8	2.8 ± 0.2	26.0 ± 1.4	32.0 ± 3.8
3.5	2.6 ± 0.15	29.5 ± 1.4	34.8 ± 3.2
3.3	2.3 ± 0.4	27.9 ± 0.8	29.5 ± 0.8
3.1	1.7 ± 0.2	25.6 ± 0.7	34.2 ± 0.8
2.1	Not detected	29.8 ± 0.6	34.2 ± 1.2
1.5	Not detected	31.9 ± 0.5	No Ct
1.0	Not detected	36.7 ± 0.6	No Ct

Data is the average of three experiments and standard deviation is shown

**Fig. 3:**
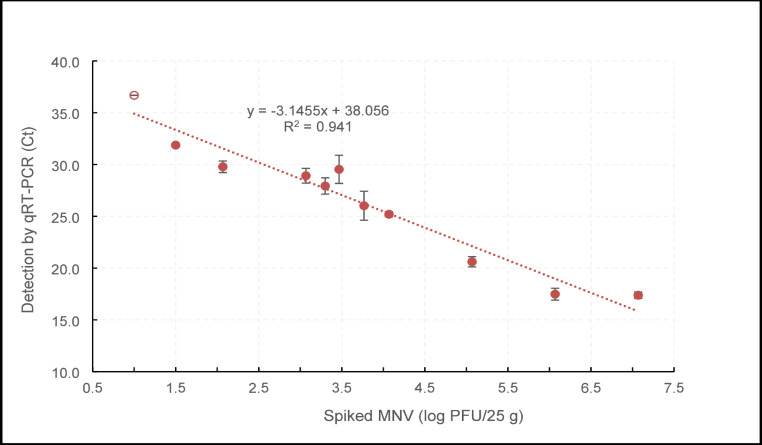
Determining the LOD by qRT-PCR from frozen raspberries. Each data point is the average of 3 replicates with error bars showing standard deviation. The empty circle demonstrates partial recovery

Detection of MNV by qRT-PCR was found to be more sensitive than cell culture. There is a linear relationship between inoculation level and Ct value (R^2^ = 0.941) until 1.0 log PFU/25 g (Fig. 3; Table 1), since at this inoculation level, only 2 out of 3 replicates produced results. This means that at Ct values higher than 36.7, the viral genome could not be consistently detected from all the replicates. Detection by qRT-PCR therefore extends about 2 log lower than cell culture.

An important consideration when comparing qRT-PCR and cell culture is that both protocols use different volumes of starting material. Considering all volumes and dilutions, each PCR reaction represented 2% of the starting material (5µL out of 100µL), while cell culture used a maximum of 1.5% per well (150µL out of 1000µL). This slight advantage in testing volume for qRT-PCR is not sufficient to explain the 2-log difference in sensitivity when compared with cell culture.

### Viral Extraction from Strawberries

**Fig. 4:**
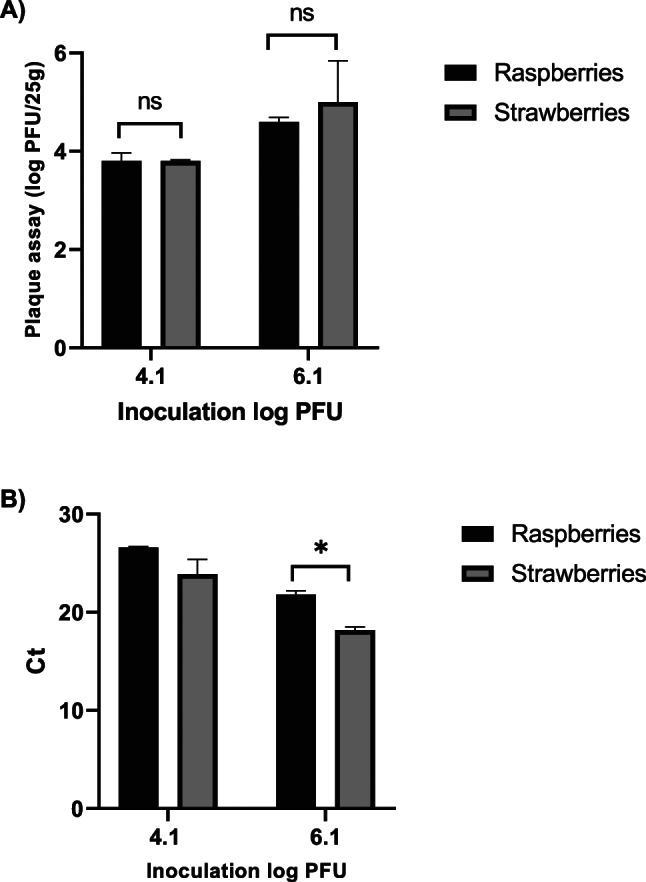
Comparison of the viral extraction from frozen raspberries versus strawberries at two inoculation levels **A** extraction of infectious MNV determined by plaque assay **B** extraction of viral genetic material determined by qRT-PCR. The data is the average of three independent experiments with error bars showing standard deviation. ns is not significant, *p ≤ 0.05 determined by t-test

We next examined the extraction efficiency for both infectious MNV and its genetic material from frozen strawberries at inoculation levels higher than the LOD. As shown in Fig. 4A and B, the viral recovery from frozen strawberries is very similar to viral recovery from frozen raspberries with no significant differences between them at medium inoculation levels and by both plaque assay and qRT-PCR. The difference is only significant by qRT-PCR at high inoculation level. This observation demonstrates that there is no marked differences between these two matrices regarding recovery of infectious MNV.

## Discussion

HuNoV is responsible for 54% of the foodborne outbreaks associated with fresh produce, and frozen berries are the major culprits (Chatziprodromidou et al., 2018). For this reason, various non-culture-based testing methods have been implemented to examine the prevalence of foodborne viruses in these high-risk commodities. Positive samples are defined by the presence of a Ct value indicating a successful amplification of the target genomic RNA. Historically these data have been employed in outbreak investigations, supply chain management, and public health protection (Jaykus et al., 2025).

In this study, we employed the ISO 15,216 method for viral isolation, which is widely accepted in the field and is often used for qualitative (presence/absence) determination for berries (Jaykus et al., 2025). We also used MNV as a surrogate for HuNoV to allow detection of infectious virus with cell culture. Overall the extraction efficiencies obtained in this study were significantly higher than the minimum requirement by ISO 15,216 (1%) and inhibition rate was negligible as for most of the tested concentrations, the delays in Ct values between 1:1 and 1:10 diluted RNA are greater than 3.3, which is an indication of 100% efficiency in qPCR (Karlen et al., 2007), also the ΔCt is greater than 2 for all the tested concentrations, which is the acceptable threshold for the ISO 15,216 method (ISO, 2017). These results were consistent between the two types of berries examined in this study.

Herein at the Ct value of 29.8 ± 0.6 or higher (i.e. 2.1 log PFU/25 g), we could not replicate the virus in cell culture. This observation is consistent with the data obtained from culturing HuNoVs in the enteroid system (Wales et al., 2023). However, we still obtained positive qRT-PCR results at Ct value of 36.7 ± 0.6 which translates into 1.0 log PFU of inoculum per 25 g. The proportion of starting material tested by each method were different but similar (1.5% for cell culture vs. 2% for qRT-PCR), yet the LOD of both methods differed by approximately 2 log. This is an indication that a significant proportion of genetic material detected by qRT-PCR does not correspond to infectious particles. Many of the RNA targets that are detected by qRT-PCR might not belong to an infectious virus, furthermore, one PFU might be comprised of an aggregate of many particles, which contain amplifiable genomes.

It is important to note the ratio between infectious and non-infectious viral particles could be different between MNV-1 and HuNoV and depend on the viral strain and the culture system that is employed for propagation. For example, the RNA: PFU ratio for MNV-1 is reported to range from 100 to 10,000 genome copies (Baert et al., 2008; Budicini et al., 2024). However, determining the infectious and non-infectious ratio for HuNoV is much more complicated as no plaque assay exists and infectivity is determined as TCID_50_. It is estimated that the minimum infectious dose for HuNoV in enteroids is around 3 log genome copies (Wales et al., 2023) but even that estimate can vary between strains (Ettayebi et al., 2024). Nevertheless the ratio between infectious and non-infectious viral particles is not drastically different between MNV and HuNoV (Atmar et al., 2014). Furthermore, ratio of non-infectious to infectious particles may change overtime on a matrix as the virus could lose infectivity but still detected by qRT-PCR (Nasheri et al.,^2021^). Therefore the LOD for qRT-PCR is always lower compared to infectivity assays.

In a Canadian surveillance study, involving berries and pomegranate arils, the HuNoV positivity rate ranged between 1.9 and 6.1% with the Ct values ranged between 33.9 and 42.2 (Steele et al., 2022), while in a European surveillance study on berries the HuNoV positivity rate ranged between 0.1 and 0.3% with Ct values ranged from 34.2 to 39.3 (Jaykus et al., 2025). In an FDA surveillance study involving frozen berries, the Ct values of the positive samples ranged between 40.75 and 49.98 (US-FDA, 2025). These Ct values are too high for determination of infectious viruses (data from this study and (Wales et al., 2023), and for effective sequencing, as it has been shown that at Ct values above 35, HuNoV sequencing cannot be consistently achieved (Yang et al., 2024). In our study, the cell culture LOD for MNV in BV-2 cells happened 6.3 Ct earlier than the qRT-PCR LOD. Even though the conditions could be different for HuNoV, it’s quite possible that qRT-PCR may generate positive results when the presence of infectious particles is questionable. Therefore interpretation of surveillance data with high Ct values, in the absence of epidemiological data, is challenging. Nevertheless, the authors acknowledge that contamination of berries with HuNoV often occurs at very low concentrations and the viral distribution is non-homogeneous, thus determination of a cut-off Ct value has not been recommended, although at elevated Ct values, a sigmoidal RT-PCR curve is required to interpret the sample as positive (Jaykus et al., 2025).

In conclusion, this study further confirms that positive qRT-PCR test results do not assure detection of infectious viruses, particularly at higher Ct values. From a risk management perspective, it could be argued that positive qRT-PCR results suggest a risk of exposure and not necessarily a risk of infection. Therefore caution should be taken in interpretation of surveillance data with elevated Ct values and in the absence of epidemiological data.

## Data Availability

No datasets were generated or analysed during the current study.
